# EPIDEMIOLOGY OF THE INVERSE COMORBIDITY OF DEMENTIA AND CANCER: THE CHALLENGE OF SELECTIVE SURVIVAL BIAS

**DOI:** 10.1093/geroni/igad104.1614

**Published:** 2023-12-21

**Authors:** Michelle Shardell, Alan Rathbun, Ann Gruber-Baldini, Jack Guralnik, Justine Golden, Dimitrios Kapogiannis, Eleanor Simonsick

**Affiliations:** University of Maryland School of Medicine, Baltimore, Maryland, United States; University of Maryland, Baltimore, Baltimore, Maryland, United States; University of Maryland School of Medicine, Baltimore, Maryland, United States; University of Maryland School of Medicine, Baltimore, Maryland, United States; University of Maryland School of Medicine, Baltimore, Maryland, United States; National Institute on Aging, National Institutes of Health, Baltimore; National Institute on Aging, National Institutes of Health, Baltimore

## Abstract

Observational studies have consistently found dementia to be inversely related to cancer, where presence of cancer predicts lower dementia risk, and presence of dementia predicts lower cancer risk. This “inverse comorbidity” relationship is of interest as an avenue to potentially identify novel intervention targets to develop for both conditions. However, it is unknown whether inverse comorbidity reflects underlying physiological mechanisms or is explained by selective survival bias. Specifically, persons with cancer are more likely to die before dementia onset than otherwise similar persons without cancer. Thus, we aimed to assess the robustness of inverse comorbidity between dementia and cancer to statistical methods that vary in how they address selective survival bias. Four discrete-time modeling methods were applied to data from the Health, Aging and Body Composition Study (n=3075; age: 70-79 years; 41.7% black; 51.5% women): (1) generalized estimating equations (GEE) assuming all missingness to be completely at random; (2) weighted independence estimating equations partly conditioned on being alive that only weight data missing for non-mortality reasons; (3) multiply-weighted GEE weighting data for all sources of missingness; and (4) marginal structural models that additionally adjust for covariates using inverse propensity score weights. Covariates included: demographics, APOEε, lifestyle characteristics, and health conditions. Methods (1)-(3) produced almost identical results: (1) Risk Ratio(RR)=0.81, 95% Confidence Interval(CI)=0.69-0.95; (2) RR=0.81, 95%CI=0.69-0.94; and (3) RR=0.81, 95%CI=0.69-0.94. Results for method (4) were slightly attenuated: RR=0.85, 95%CI=0.72-0.99. Inverse comorbidity was consistently observed and robust to statistical handling of missing data/mortality. Future research aims to identify mechanisms of inverse comorbidity.

